# The Environmental Impact of Cambodia's Ancient City of Mahendraparvata (Phnom Kulen)

**DOI:** 10.1371/journal.pone.0084252

**Published:** 2014-01-08

**Authors:** Dan Penny, Jean-Baptiste Chevance, David Tang, Stéphane De Greef

**Affiliations:** 1 School of Geosciences, The University of Sydney, Sydney, New South Wales, Australia; 2 Archaeology and Development Foundation, London, United Kingdom; University of Toronto Mississauga, Canada

## Abstract

The Khmer kingdom, whose capital was at Angkor from the 9^th^ to the 14^th^-15^th^ century, was founded in 802 by king Jayavarman II in a city called *Mahandraparvata*, on Phnom Kulen. Virtually nothing more is known of *Mahandraparvata* from the epigraphic sources, but systematic archaeological survey and excavation have identified an array of cultural features that point to a more extensive and enduring settlement than the historical record indicates. Recent remote sensing data have revolutionized our view, revealing the remains of a city with a complex and spatially extensive network of urban infrastructure. Here, we present a record of vegetation change and soil erosion from within that urban network, dating from the 8^th^ century CE. Our findings indicate approximately 400 years of intensive land use, punctuated by discrete periods of intense erosion beginning in the mid 9^th^ century and ending in the late 11^th^ century. A marked change in water management practices is apparent from the 12^th^ century CE, with implications for water supply to Angkor itself. This is the first indication that settlement on *Mahendraparvata* was not only extensive, but also intensive and enduring, with a marked environmental impact.

## Introduction

Angkor was the vast low-density capital of the Khmer Kingdom, from the early 9^th^ to the mid-14^th^/15^th^ centuries of the Common Era (CE). At its peak, Angkor sprawled over nearly 1000 km^2^ [1] and may have housed more than three quarters of a million people [2], [3]. The primary administrative centre in a kingdom that dominated most of mainland Southeast Asia by the 11^th^ century CE, Angkor was the largest preindustrial city on Earth and remains the world's largest archaeological site.

**Figure 1:**
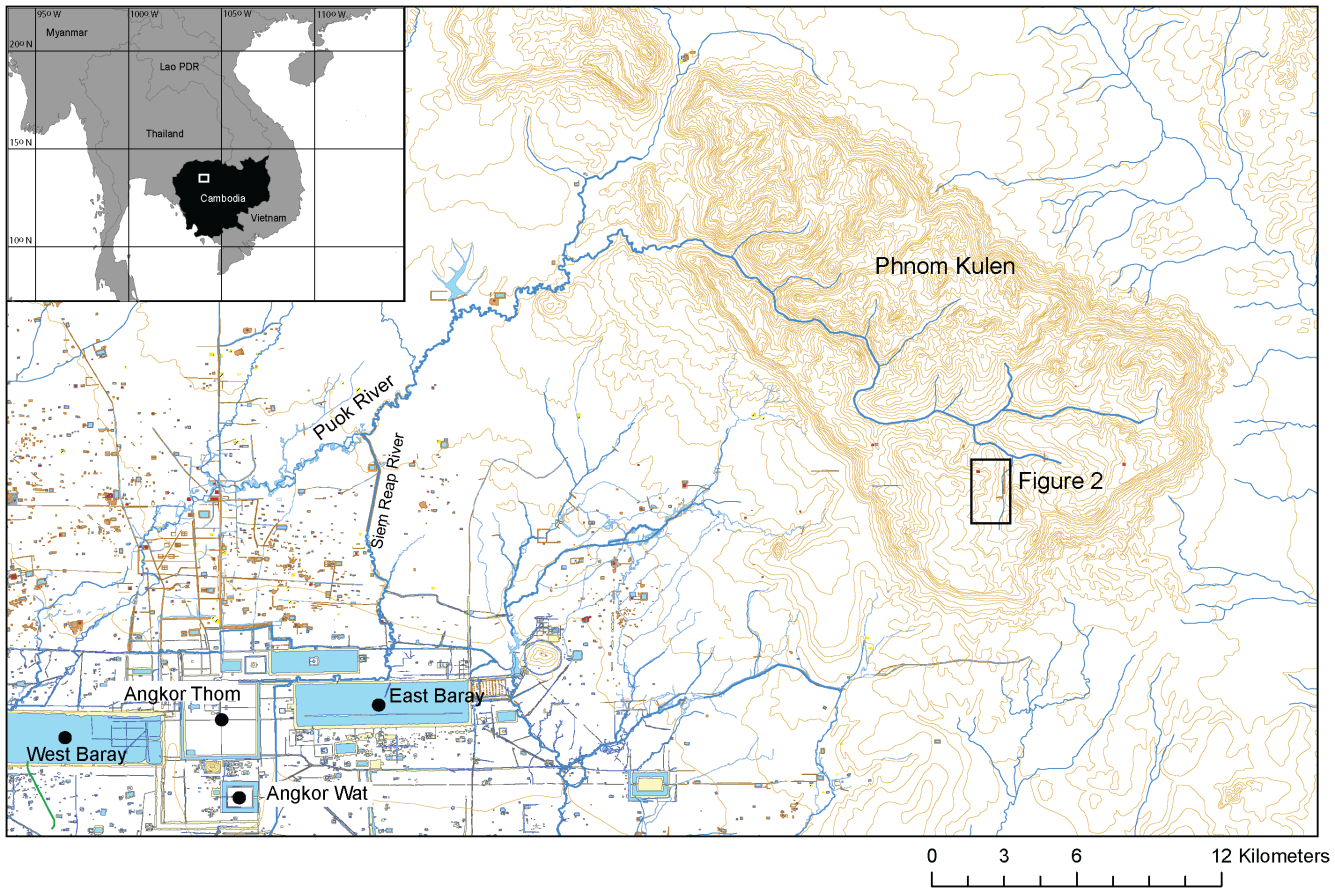
Map of central/northern Angkor and Phnom Kulen (Mahendraparvata). Contours in 10[17] and [1], topography and hydrology data from the Japan International Cooperation Agency (JICA).

The Angkor period is commonly understood to start in 802 CE with the proclamation of Jayavarman II as the *chakravartin* (universal-king) from a location in the Kulen mountains (Phnom Kulen), overlooking the vast alluvial plain where Angkor would begin to emerge in the following centuries (Figure 1). In doing so, Jayavarman confirmed himself as the great unifier; drawing Cambodia's disparate polities together under the first ‘god king’ and establishing the Khmer state and the basis of its empire. Phnom Kulen was known as *Mahendraparvata*; “the hill of the great Indra”.

The extant history of *Mahendraparvata* is based on several inscriptions, the most well-known being an 11^th^ century CE inscription (K.235) found at the Sdok Kak Thom temple, in eastern Thailand [4]. The inscription, dated to 1052 CE, outlines the lineage of a private family serving successive Khmer Kings for two and a half centuries, the first mentioned being Jayavarman II. Much attention has been given to the veracity of the data contained in the inscription, and it is widely considered either questionable, or a fiction [5]. The urban form of *Mahendraparvata* remains poorly understood, though the larger monuments, cave sites, reservoirs and ceramic kilns in the Kulen have been known, if imperfectly, for many years [6], [7], [8], [9], and recent work by one of us [10] in recording and dating these features has made a significant contribution.

A recent LiDAR mission over Phnom Kulen [11] has revealed an extraordinary array of cultural features beneath the forest canopy. The implications of this discovery are still under study, but it seems certain that Phnom Kulen is part of a long and complex history of the Angkor region, stretching back millennia [12], [13], [14], [15], [16]. In particular, the LiDAR indicates so much construction that large areas of the central plateau of the Kulen would have been extensively or completely deforested, just as they were on the Angkor plain to the south [17].

This paper presents a sedimentological and palaeobotanical analysis of a sediment core from one of the ancient reservoirs that make up the extensive archaeological landscape of the Kulen. Our aim is to determine if evidence of intensive land-use exists in these sedimentary archives, and to establish an independent, isotopically (^14^C) dated chronology for the occupation and abandonment of *Mahendraparvata*.

### Phnom Kulen

Phnom Kulen is an elongated plateau measuring ∼15 km (SW-NE) to ∼25 km (NW-SE) situated approximately 40 km to the northeast of central Angkor [18]. Rivers have eroded the Jurassic and Cretaceous-aged sandstone to form a ‘bowl’ or ‘amphitheatre’ plateau that opens to the northwest, with the rim of the southeast part of the plateau rising abruptly to a maximum of 490 m above the flat Angkorian plain. The margin of the plateau is marked on all sides by a steep escarpment [19]. Precipitation on the plateau is higher on average (1854 mm/yr) than the Tonle Sap floodplain (circa 1183 mm/yr) and on the high plain at Banteay Srei 20 km to the north east of Angkor (932 mm/yr), with precipitation at the lakeshore line and high plain only 64% and 50% of the mountain precipitation, respectively [8], [20].

Phnom Kulen was, until recently, heavily forested, although the historical extent of deforestation is entirely unknown [21]. Today, most of the forest on the southern part of the plateau has been lost to swidden agriculture and cashew nut plantations. Phnom Kulen's main historical and geographical significance lies in its role as Angkor's source of water [20]. The sandstone of the Kulen is permeable, meaning that peak seasonal rainfall is stored and slowly released during the course of the year, ameliorating the sharply seasonal climate. Boulbet [8] claimed that rainfall began in the Kulen before the rest of the Siem Reap plain, during the normally hot and dry months of March-April, although we note, anecdotally, that this pattern has changed in recent years.

An integral part of Phnom Kulen's archaeological landscape is a number of dams, claimed by Van Liere [22] (following [9]) to be the greatest concentration of reservoirs in the Khmer realm. In fact, only five major dams (*Thnal*) have been confirmed by ground surveys so far, and many other similar sites have different characteristics and, presumably, different functions [10]. For the most important of them, the dams were earthen dykes, often faced by laterite slabs on the reservoir side, and without apparent gated structures. Van Liere [22] interpreted the absence of structures to control the release of impounded water as evidence for their use as flood retardation devices rather than water storage for agriculture, for which he claims there was insufficient capacity.

However, apart from its significant but imperfectly understood early history, we are left with few details about *Mahendraparvata* during the Angkor period. Indisputably, the foothill region acted as the supply of sandstone to the temples in the Angkorian capitals, and several quarries have been discovered in the southeast of the massif [8], [23]. Additionally, more than 30 brick temples have been discovered on the plateau itself, the first of which were recognised in 1883 [6], [10], [24], [25], [26], [27], as well as rock shelters, river bed sculptures, and rock paintings [10], [28], [29]. Furthermore, Phnom Kulen represents one of the most significant ceramic production centres within the Khmer kingdom [28], [30]. Recent exploratory archaeological work on kilns located on top of Thnal Mrech, near Anlong Thom village, place the operation of two different kilns from the 10^th^ to 11^th^ centuries (ALK01) [31] and from the 11^th^ to the 13^th^ centuries CE (TMK 02) [32].

## Materials and Methods

### Site Description

**Figure 2:**
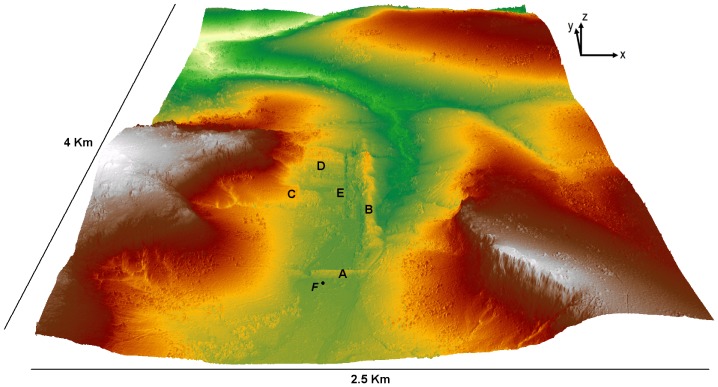
Lidar digital terrain model of Phnom Kulen Plateau centered on Thnal Mrech dyke. Lidar digital terrain model of the main valley of Phnom Kulen Plateau with 1× vertical exaggeration. A: Thnal Srae Thbong; B: Thnal Mrech; C: NS cut bedrock; D: EW extraction lines; E: channel; F: Sediment core sampling (Imagery: Stéphane De Greef/Archaeology and Development Foundation).

Prior to the recent LiDAR mission two main pieces of hydrological infrastructure were known in this area. The East-West orientated *Thnal Srae Thbong* (Southern Ricefields Dyke) blocks the main river running south to north, in the catchment that drains most of southern plateau. The *Thnal Srae Thbong* dike, used as a dam and creating a large reservoir upstream as well as a road to cross the valley, is associated to the *Thnal Mrech* (Pepper Dyke), a massive N-S oriented dike, upon which are several known ceramic kilns sites. Both ground surveys and LiDAR results have shown that no physical connection exists between these two dykes (Figure 2).

The dykes themselves are massive - the *Thnal Mrech* dyke is almost 1 km in length, 60 m wide, and 5–12 m high. *Thnal Srae Thbong dyke*, faced with laterite masonry on the northern (reservoir) side of the dyke, measures 280 m long, 35 m wide and 3.60 m high [9], [10], [33]. The *Srae Thbong* dyke (as well as *Thnal Dac*, a similar blocking dyke east of Anlong Thom village), has a large western breach that was the result of bombing by Lon Nol forces in 1971–73 [10]. This reflects the critical strategic value of these reservoirs as a source of water for the alluvial plain to the south.

LiDAR data reveal that the western side of the valley, north and downstream of the *Thnal Srae Thbong*, has been cut into bedrock and alluvium (Figure 2, C), strictly paralleling the *Thnal Mrech* dyke which forms the eastern side of the valley. Smaller E-W oriented features in the northern part of artificial valley (Figure 2, D) [10], [33] indicate the leveling of bedrock in the area in order to create this reservoir, and presumably to use the quarried material (sandstone, laterite, soil) in the extensive monumental construction program. This could imply that a large reservoir was planned, but never completed. A small channel on the eastern side of the blocking *Thnal Srae Thbong*, revealed for the first time in the LiDAR data (Figure 2), may have been used to discharge water to the eastern side of *Thnal Mrech* while construction of the downstream reservoir was underway. Alternatively, the existing *Thnal Srae Thbong* reservoir may have been a temporary construction, blocking flow down the valley to permit the excavation and construction of the larger reservoir further to the south, which would eventually supersede it. Confirmation will require further field studies [10], [22], but we may consider the entire valley to be a single reservoir, only one part of which was actually operational.

Boulbet [8] mapped a northern dike running E-W across the valley, *Thnal Choeng* (North Dike), but it is difficult to verify its existence today in the field or with the LiDAR data. *Thnal Choeng* may be related to a small dyke associated with an ancient field system, or the northern edge of the quarried area in the north of the valley. In any case, it is a much smaller feature that is not functionally related to water management in the valley, and is not considered further here.

The reservoir south (upstream) of *Thnal Srae Thbong* is the focus of our study. The reservoir is now completely colonised by a floating herb swamp and woody swamp forest vegetation. Dominant canopy species recorded by us in 2010 are *Macaranga triloba* (Euphorbiaceae) and *Eugenia* (*Syzygium) syzygioides* (Myrtaceae). *Melastoma villosum* (Melastomataceae) is the dominant shrub on the swamp surface, and these are commonly festooned with the epiphytic ferns *Stenochaena palustris* (Blechnaceae) and, in areas with no swamp forest canopy, *Lygodium microphyllum* (Lygodiaceae). Fern taxa, in particular, are very diverse. Sedges (Cyperaceae) and grasses (Poaceae) are common throughout, with Cyperaceae co-dominant with *Alocasia macrorrhizos* (Araceae) in sheltered areas at the margins of the swamp. In open water areas *Nympoides* spp. (Menyanthaceae) are co-dominant with *Utricularia aurea* (Lentibulariaceae). The vegetation communities on the dykes themselves, and in the surrounding catchment, are typically dry- to mixed-deciduous forests dominated by the Dipterocarpaceae (*Dipterocarpus* spp., *Hopea* spp.. *Shorea* spp.) [8], [34].

### Sampling

Sediment cores were extracted from the reservoir south of *Thnal Srae Thbong* in June 2009. Coring was performed using a rope-operated Kullenberg-type piston corer [35], using 4 m lengths of 60 mm diameter PVC tubes. A wooden coring tower was erected on the floating vegetation mat in order to anchor the piston relative to the swamp surface. The core was taken approximately 30 metres upstream of the dam wall, close to the centre of the flooded valley (13° 31′ 31.1154″ N; 104° 9′ 24.3″ E; WGS84 Zone 48N). Two replicate cores (sample code TMR/A 1.78 m in length and TMR/B 2.12 m in length) were taken from this site to ensure that a representative sample had been obtained. Cores were capped and sealed on recovery, and shipped to the University of Sydney where they were stored at 3°C. Any material remaining after the completion of analytical work was archived in the University's cool storage facility.

### Laboratory Anal*y*ses

Cores were split longitudinally and sub-sampled at 10–50 mm intervals with depth down core. Three samples of macroscopic unburnt wood (10–65 mm in diameter) were extracted from TMR/B and submitted for accelerator mass spectrometry radiocarbon dating, with a standard acid-alkali-acid pre-treatment. Radiocarbon ages were calibrated to years CE using CALIB 6.0 [36] using the IntCal04 northern hemisphere calibration dataset [37] and reported following Stuiver and Polach [38].

Measurements of volumetric magnetic susceptibility (κ), which we anticipate reflects changes in sediment type and/or source [39], were taken using a Bartington MS2E sensor attached to a Bartington MS3 meter. Measurements were made at 1 cm intervals. The relative abundance of organic carbon in the reservoir sediment was determined by loss on ignition, following [40], [41] (ie: LOI_550_, 4 hrs). Mineral particle size analysis was performed in order to quantify sediment texture through the sequence. Between 0.4 to 1.0 g of sediment was oxidized in 35% w:w H_2_O_2_ in order to remove organic matter, deflocculated with (NaPO_3_)_6_ and analysed using a Malvern Mastersizer 2000 laser diffraction spectrometer. No samples were taken for particle size analysis from the fibrous peat sections of the core because mineral concentrations were extremely low and full oxidisation of plant matter judged to be ineffective.

Plant microfossils (pollen and spores) were extracted from 40 sub-samples, taken at 30–100 mm intervals with depth. 1.0 cm^3^ sub-samples were treated with sodium hexametaphosphate dispersant and wet sieved at 125 µm, with the <125 µm proceeding to digestion, which followed standard protocols [42]. Microfossils were identified and tallied using light microscopy at 400–1000× magnification. Pollen/spore counts were converted absolute abundances following Stockmarr [43] and plotted using C2 software [44]. Zonation of the microfossil assemblage was determined through stratigraphically constrained cluster analysis, using Cluster 3.0 [45]. All samples are archived at the University of Sydney.

## Results

### Sedimentology

**Figure 3:**
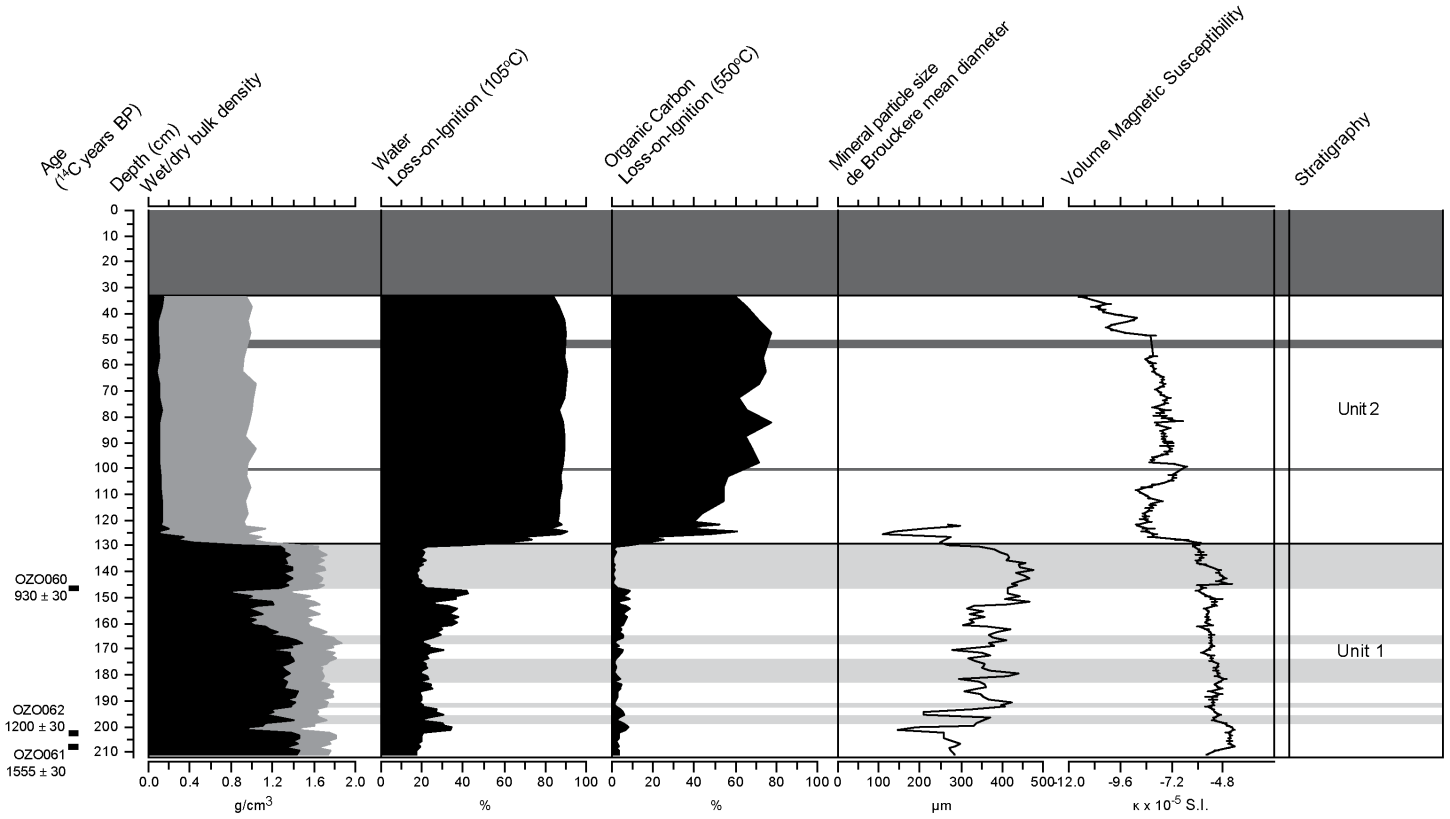
Sedimentology of core TMR/B. Plot of wet (grey)/dry (black) bulk density, loss-on-ignition at 105°C and 550°C, DeBroukere mean diameter, volumetric magnetic susceptibility and stratigraphic units for core TMR/B. Light gray shading represents sand beds. Dark gray shading represents no data. See text for descriptions.

Two distinct units can be identified from core TMR/B (Figure 3). *Unit 1* (212-129 cm depth in core) is primarily siliciclastic, characterised by low organic content (under 10% of dry weight), low moisture content (15–40% of wet weight) and relatively high mineral bulk density (average 1.67 g/cm^3^). It is predominantly sand to loamy sand, dark gray (10YR 4/1) to very dark gray (10YR 3/1) in colour [46]. There are few small (<10 mm) wood and charcoal pieces throughout the matrix, becoming common at 171 cm depth.

**Figure 4:**
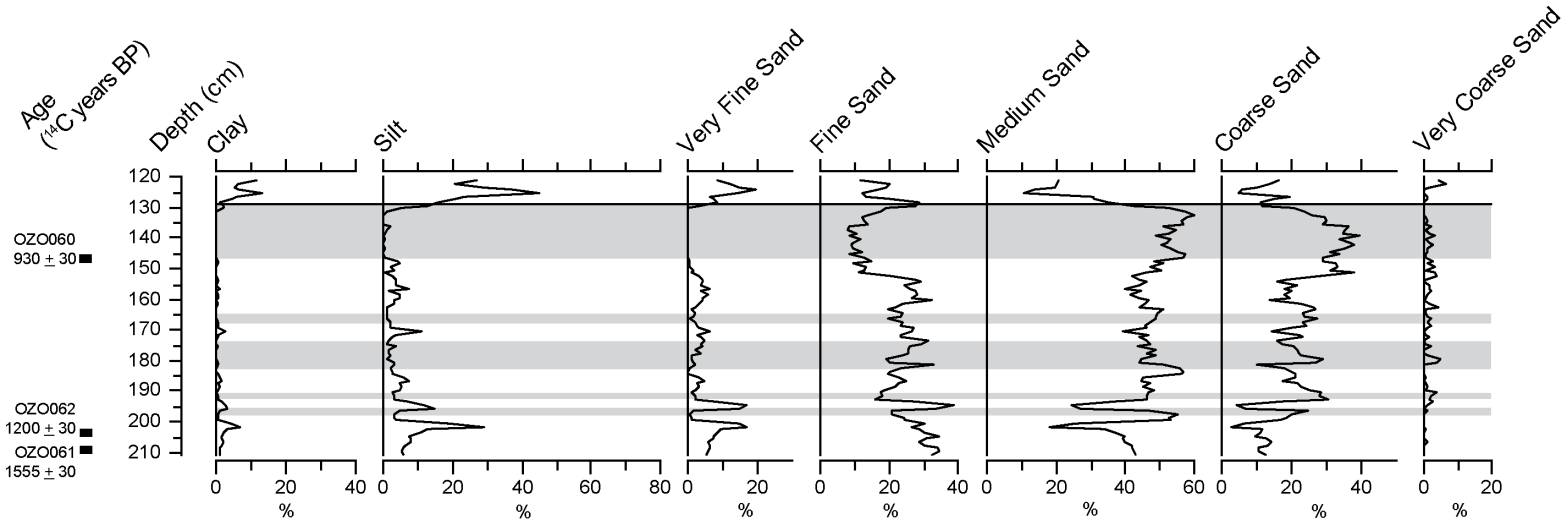
Mineral particle size analysis of core TMR/B. Gray shading represents sand beds. Particle size classification follows the Wentworth classification scheme [56].

This unit is interbedded with five beds of medium-to-coarse sand (196–198, 191–193, 174–183, 165–168 and 129–147 cm depth in core, respectively). The largest of these, located at 147-129 cm depth, is 18 cm in thickness and is composed of 99.1% sand (Figure 4). These beds are relatively low in organic carbon (an average of 1.5% of dry weight), and magnetic susceptibility values are negative and lower than the rest of Unit 1.

*Unit 2* (129-0 cm depth in core) is a black (10YR 2/1) fibric to humic peat. On average, organic carbon values are high relative to Unit 1 (an average of ∼50% dry weight). Bulk density (an average value of <1 g/cm^2^) and magnetic susceptibility (an average value of -8.26×10^−5^ SI) values are low relative to Unit 1 (1.67 g/cm^3^ and −5.25×10^−5^ SI, respectively) reflecting a very small mineral component. Macroscopic plant remains are common throughout, including large fragments of intact, saturated wood. The upper 32 cm of the core was extremely fluid, representing a portion of the sediment-water interface beneath the floating vegetation mat, and was not sampled as we presume it to have been mixed during transportation.

### Chronology

AMS radiocarbon dates for wood samples taken from TMR/B are presented in Table 1. The two wood samples from close to the base of core TMR/B (OZO062, 203 cm depth, and OZO061, 208.5 cm depth) returned radiocarbon ages of 1200±30 and 1555±30 ^14^C years BP, calibrating to the 5-6^th^ and 8-9^th^ century CE, respectively. A wood sample taken from the base of the uppermost sand bed in Unit 1, which marks the abrupt boundary between Units 1 and 2 (OZO060, 146.5 cm depth), returns a radiocarbon age of 930±30 years BP, calibrating to 11-12^th^ century CE.

**Table 1:** Results of AMS radiocarbon dating of wood samples from core TMR/B.

Code	Depth (cm)	δ(^13^C) ‰	pMC1±1σ	^14^C years BP±1σ	Calibrated age CE (2σ 95.4% probability)
OZO060	146.5	−23.9±0.1	89.09±0.32	930±30	1025–1168 [1.00]
OZO062	203	−26.1±0.1	86.11±0.31	1200±30	712–745 [0.065339]
					767–895 [0.922011]
					925–936 [0.012651
OZO061	208.5	−29.0±0.1	82.39±0.27	1555±30	425–570 [1.00]

Ages are calibrated to years CE at 2σ, or 95.4% confidence. Figures in square brackets indicate the proportion of the probability range represented by each intersection of the probability curve. Measured σ^13^C values solely relate to the graphite derived from the material used for radiocarbon measurement.

s

### Palynology

**Figure 5:**
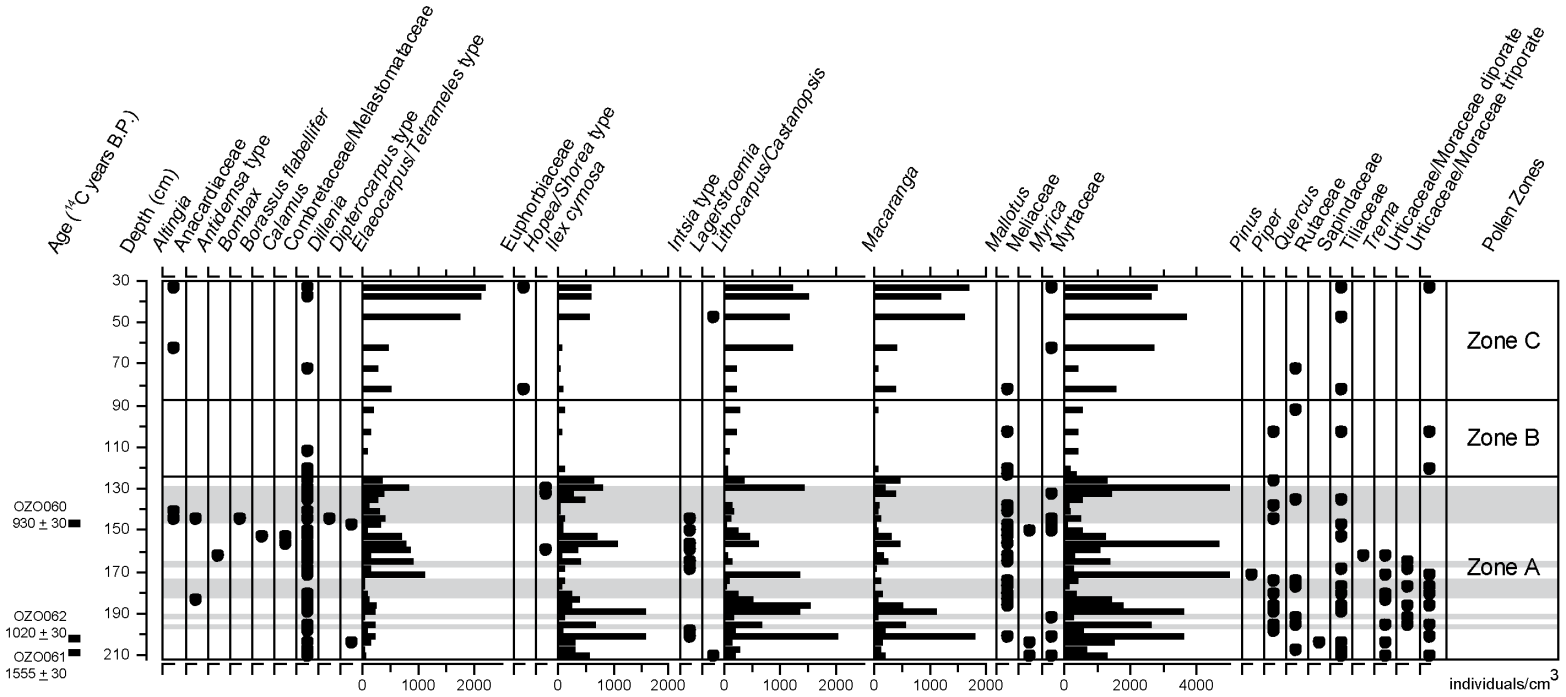
Pollen results (trees and shrubs). Concentration of pollen (individuals per cm^3^ of sediment) from trees and shrubs, plotted against depth/age, for core TMR/B. Closed circles represent rare taxa that did not exceed 5% of the total microfossil assemblage in any sample. Light gray shading represents sand beds. Pollen zones based on classification of the whole assemblage.

**Figure 6:**
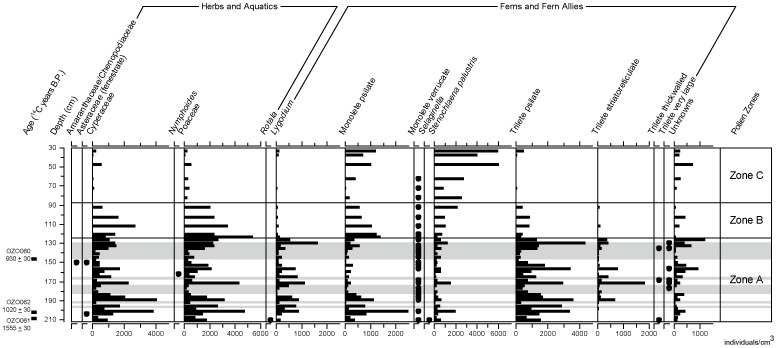
Pollen results (herbs, ferns). Concentration of pollen (individuals per cm^3^ of sediment) from herbaceous plants (dryland, wetland and aquatic), ferns, and ‘fern allies’ (*Selaginella*, Lycopodiopsida), plotted against depth/age, for core TMR/B. Closed circles represent rare taxa that did not exceed 5% of the total microfossil assemblage in any sample. Light gray shading represents sand beds. Pollen zones based on classification of the whole assemblage.

45 known pollen and spore taxa, representing 33 families (excluding the ‘monolete’ and ‘trilete’ fern spore groups), were identified from 40 samples taken from core TMR/B. In total, 4416 individuals were identified, with an average of 110 individuals per slide. Three distinct palynological zones can be identified using stratigraphically constrained cluster analysis of the entire assemblage – Zone A (2.12-1.25 m depth), Zone B (1.25-0.875 m depth) and Zone C (0.875-0.3 m depth). These are shown in Figures 5 and 6.

## Discussion

### Flooding of the Valley

There is no evidence of pre-reservoir floodplain soil in core TMR/B. The base of Unit 1 lacks the Fe or Mn mottling typical of wet/dry soils in the seasonal tropics, shows no evidence of pedogenesis (pedal structure, root penetration) or evidence of coarse cross-bedding (bedload) or episodic, normally-graded fining-up sequences (flood pulse). Rather, Unit 1 is massively bedded and well-sorted (mean σ 212 to 197 cm = 2.25 µm) with a unimodal and leptokurtic particle size distribution characterized by a long and thin fine tail, indicating sedimentation in water. We take the absence of a basal floodplain unit to indicating that the core barrel did not penetrate through the valley fill. Consequently, we are unable to date the flooding of the valley following the construction of the blocking dyke. OZO061 implies a *minimum* age for the reservoir of 5-6^th^ centuries CE (425–570 CE; Table 1). However, there is no archaeological evidence for settlement anywhere on the Kulen at that time, and we therefore consider the wood sample from which this age is measured to be older than the sediment in which it was interred. This is perhaps a result of the clearing of primary forest and/or reworking of wood fragments associated with the initial construction of the dyke. OZO062 returned a calibrated age of 767-895 CE (representing 0.92 of the probability distribution at 2σ; Table 1), which is closely coherent with existing archaeological evidence for large-scale settlement in Phnom Kulen. This age was measured from a wood fragment recovered 9 cm above the base of the core, which was therefore interred some time after the construction of the reservoir. Linear interpolation between the mean of the 2 σ calibrated age range for OZO062 and OZO060 predicts a basal age for the core (212 cm depth) of c. 780 CE. Our hypothesis, therefore, is that the reservoir was constructed no later than the mid-late 8^th^ century CE.

### Vegetation of Zone A, 8^th^-11^th^ Centuries

The arboreal pollen assemblage deposited during this period (Zone A in Figure 5, equivalent to stratigraphic Unit 1) is dominated by swamp forest taxa, representing about 40% of the total microfossil assemblage. The most abundant of these is Myrtaceae, most probably *Syzygium* (*Eugenia) syzygioides*, which is very common on the swamp surface presently. Other notable pollen types include *Ilex cymosa* (swamp holly), which favours wet dense forests [47], and *Macaranga*, most probably *Macaranga triloba*. Fern spores are extremely common in sediment deposited during this period, though only *Lygodium* can be identified securely (Figure 6).

The overwhelming dominance of swamp-forest and wetland pollen in the assemblage makes interpretation of dryland vegetation on the surrounding slopes problematic. The *Elaeocarpus*/*Tetrameles* pollen group may, in whole or in part, derive from dryland forests, but *Elaeocarpus* can be recruited into swamp forest in Cambodia [48]. *Tetrameles*, however, occurs in semi-evergreen forest, but it is impossible to be more specific without resolving the problematic taxonomy of these pollen types [49]. The same is true of the *Lithocarpus/Castanopsis* pollen group, as species such as *Lithocarpus polystachyus* are known to occupy periodically inundated sites [48] and may have been recruited into littoral swamp forest communities. Despite the occurrence of pollen from common dryland taxa (such as *Dipterocarpus*), there is no unequivocal dryland forest signal in the pollen record, and only one occurrence of an economic tree (*Borassus flabellifer*, four individuals recorded at 153 cm depth, an interpolated age of middle 11^th^ century) [50].

### Soil Erosion

The episodic deposition of medium-to-coarse sand into the reservoir, beginning in the mid 9^th^ century (198 cm) and ending in the late 11^th^ century (129 cm), indicate discrete periods of significant soil mobilisation within the catchment, either from the surrounding low hills or from the dyke itself. It is impossible to quantify reliably the volume of sand deposited during these episodes without more extensive sampling in the reservoir but, given the thickness of the coarse sand units in cores from the mid-valley, they may represent very large volumes of material, and significant periods of soil erosion. We can only speculate as to the cause of these episodes. They may relate to particularly intense summer monsoon rainfall events, high-energy floods related to the release of water from smaller upstream reservoirs, or periods of clearing on slopes proximal to the reservoir related to slash-and-burn agriculture or building activity. These episodic periods of erosion might also relate, in part, to the construction of a carved sandstone canal (*O Tuk Lik*) higher in catchment, intended to bring water south, over the escarpment edge and down to the Beng Mealea region.

Whatever the specific cause of these sand deposits, the recent discovery of archaeological features beneath the remaining forest canopy on Phnom Kulen suggests that historical land use in the area was intense and very extensive, and that clearing of primary forest must have been very extensive. Our data suggest that settlement on Mahandraparvata was not only spatially extensive but temporally enduring, and sufficiently intensive to trigger extensive soil mobilisation within the catchment over approximately 250 years from the middle of the 9^th^ century CE.

### Vegetation of Zone B and C; Post 12^th^ Century CE Abandonment and Swamp Succession

The abrupt transition from lithogenic to biogenic sediment at 129 cm depth (an interpolated age in the early to mid 12^th^ century) indicates the colonisation of the reservoir surface by herbaceous swamp. These changes are broadly coincident with the construction program of Suryavarman II, before the zenith of Angkor's monumental construction under King Jayavarman VII. At least one ceramic kiln within the Thnal Mrech dyke was still in operation at that time [32].

The boundary between these two units is accute and punctuated by the last and most significant period of soil erosion, suggesting that this change in sediment type was not driven by a shift in sediment source as the reservoir terrestrialised. Rather, this abrupt transition may be viewed as a change in the way the reservoir was managed, analogous to the abandonment of temple moats in Angkor itself [51]. Change in the function or operation of the reservoir may reflect changes in population or land use on *Mahendraparvata*, or may be a response to changing priorities in water management within Angkor, whence the water from *Thnal Mrech/Srae Thbong* would eventually flow. We hypothesise that construction/excavation of the northern *Thnal Mrech/Thnal Choeng* reservoir was abandoned at this time. The colonization of the *Srae Thbong* reservoir is broadly coincident with changes in hydrology of the West Baray [52] and East Baray [53], [54] at Angkor, and may point to a broader change in water management throughout the region.

Increases in Poaceae and Cyperaceae pollen at the base of zone B (c. early-mid 12^th^ century) may reflect the initial, relatively rapid colonization of the reservoir surface by herbaceous plants and ferns (reflected in the data set by the trilete and monolete spore groups). The near-exponential rise in the concentration of *Stenochlaena* spores through Zones B and C (particularly above 0.875 m depth in the core, or c. middle to late 14^th^ century CE) and the concomitant decline in the abundance of Cyperaceae and Poaceae pollen during that period, may reflect the establishment of swamp forest trees proximal to the core site (that is, mid channel). This is a successional change within the reservoir itself, presumably related to the development of a floating vegetation mat substantial enough to support large woody species. Trees provide a substrate for the epiphytic *Stenochlaena* and shade-out grasses, sedges and ground ferns such as *Lygodium* [55]. Prior to this time, then, swamp forest communities may have existed as a fringe of vegetation in the margins of the reservoir, periodically inundated during the wet season. By the mid 16^th^ century the flora growing on the site is broadly comparable to its modern flora.

## Conclusion

This paper presents stratigraphic and palaeobotanical data from a large Angkor-period reservoir on the central plateau of the Kulen Hills. We find that the valley was flooded no later than the mid to late 8^th^ century CE, but the age of the reservoir remains inconclusive. The reservoir operated for perhaps 400 years, and during that time several episodes of intense erosion and deposition occurred within the catchment. We take this to indicate intensive land use, in strong agreement with recent evidence for very extensive patterns of occupation in the Kulen, and particularly within the *Thnal Mrech/Srae Thbong* catchment. The last and largest episode of erosion occurred in the late 11^th^ century, and this event reflects some change in the operation or management of the reservoir. The reservoir was colonised by vegetation from the 11-12^th^ centuries CE, perhaps reflecting a population decline on the plateau at that time. These events may also be linked to changes in water management and settlement organization in Angkor during the 12^th^ century. Patterns of vegetation change on the site from that time appear consistent with gradual vegetation succession as the herbaceous and, later, swamp forest community matured. During 2010 swamp forest communities were being aggressively cleared from the ancient reservoirs, part of a broader pattern of forest clearance in the Kulen Hills that, arguably, mirrors historical events described here.
